# Recent Advances in Diabetic Kidney Diseases: From Kidney Injury to Kidney Fibrosis

**DOI:** 10.3390/ijms222111857

**Published:** 2021-11-01

**Authors:** Peir-Haur Hung, Yung-Chien Hsu, Tsung-Hsien Chen, Chun-Liang Lin

**Affiliations:** 1 Department of Internal Medicine, Ditmanson Medical Foundation Chia-Yi Christian Hospital, Chiayi 600566, Taiwan; dtmedg3@yahoo.com.tw (P.-H.H.); cych13794@gmail.com (T.-H.C.); 2 Department of Applied Life Science and Health, Chia-Nan University of Pharmacy and Science, Tainan 717301, Taiwan; 3 Department of Nephrology, Chang Gung Memorial Hospital, Chiayi 613016, Taiwan; libra@cgmh.org.tw; 4 Kidney and Diabetic Complications Research Team (KDCRT), Chang Gung Memorial Hospital, Chiayi 613016, Taiwan; 5 School of Traditional Chinese Medicine, College of Medicine, Chang Gung University, Taoyuan 333423, Taiwan; 6 Kidney Research Center, Chang Gung Memorial Hospital, Taoyuan 333423, Taiwan; 7 Center for Shockwave Medicine and Tissue Engineering, Chang Gung Memorial Hospital, Kaohsiung 833253, Taiwan

**Keywords:** diabetic kidney disease, inflammation, albuminuria, fibrosis, glomerulosclerosis

## Abstract

Diabetic kidney disease (DKD) is the leading cause of chronic kidney disease and end-stage renal disease. The natural history of DKD includes glomerular hyperfiltration, progressive albuminuria, declining estimated glomerular filtration rate, and, ultimately, kidney failure. It is known that DKD is associated with metabolic changes caused by hyperglycemia, resulting in glomerular hypertrophy, glomerulosclerosis, and tubulointerstitial inflammation and fibrosis. Hyperglycemia is also known to cause programmed epigenetic modification. However, the detailed mechanisms involved in the onset and progression of DKD remain elusive. In this review, we discuss recent advances regarding the pathogenic mechanisms involved in DKD.

## 1. Diabetes Mellitus and Diabetic Kidney Disease

Currently, more than 400 million people live with diabetes mellitus (DM) globally. This number is expected to increase to 600 million by 2035 [1]. DM affects people of all ages, irrespective of sex, ethnicity, education level, or financial status [2]. Among DM patients, 20% may progress to diabetic kidney disease (DKD) [3], which is known to be influenced by both genetic and environmental factors and induced by microvascular and macrovascular changes, including accumulation of extracellular matrix and hypertrophy and fibrosis of the kidney glomeruli and interstitium [4,5]. At onset, DKD patients typically show microalbuminuria symptoms, with 30 to 300 mg of albumin excreted per day; this gradually develops into macroalbuminuria, with more than 300 mg of albumin excreted per day at later disease stages [6]. The hazard ratio for all-cause mortality in DKD patients with macroalbuminuria is reported to be 1.83, compared to 1.46 for patients with normoalbuminuria [7]. Overall, a complex interplay between metabolic processes, epigenetic and nonepigenetic mechanisms, and transcriptional regulation is involved in the development and progression of DKD, and only in the last few years have potential drugs been identified, such as sodium-glucose cotransporter 2 (SGLT2) inhibitors that can act effectively against hypoglycemia and improve kidney outcomes [8,9]. In addition, endothelin-1 (ET-1) has been associated with vasoconstriction, kidney injury, mesangial hyperplasia, glomerulosclerosis, fibrosis, and inflammation, and thus endothelin receptor antagonists have been proposed as potential treatments for DKD [10].

## 2. Influence of Hyperglycemia on Diabetes-Mediated Cellular Alterations

Due to unregulated expression of glucose transporters, high levels of extracellular glucose will ultimately increase intracellular glucose concentration [11], resulting in the shunting of glucose to the fructose 6-phosphate and hexosamine metabolic pathways [12]. Therefore, hyperglycemia often increases the production of advanced glycation end products (AGEs) and reactive oxygen species (ROS), which are closely associated with the development of DKD. AGEs are formed by nonenzymatic glycation reactions between reducing sugars and amino acids, lipids, or DNA, and are associated with high levels of ROS production [13]. ROS are produced during mitochondrial oxidative metabolism and after exposure to xenobiotics and cytokines, through reactions catalyzed by NADPH oxidase, nitric oxide synthase, and xanthine oxidase [14], and excessive ROS will cause oxidative stress and cell damage. Earlier studies have demonstrated that limiting the production of AGEs and ROS effectively slows the progression of DKD [15]. In addition, ROS are known to activate the Janus kinase signal transducers and activators of transcription (JAK-STAT) pathway, and experiments in a mouse diabetes model showed that the selective expression of JAK2 in glomerular podocytes increased the functional and pathological characteristics of DKD [16]. Moreover, in the kidney tissues of DKD patients, significantly increased expression levels of multiple JAK-STAT family members have been observed [17].

Under high-glucose conditions, ROS are produced at high levels, and this can cause diabetic complications [18,19]. The excessive production of ROS is primarily attributed to the activation of electron transport chains and the electron leakage of NADH dehydrogenase in mitochondria [20]. Loss of mitochondrial control impacts renal health since the mitochondria are the major source for ROS formation, apoptosis, and metabolism. Such excess ROS production is known to cause DNA damage [21], and this in turn induces poly-ADP ribose polymerase-1 (PARP-1) activation to inhibit glyceraldehyde 3-phosphate dehydrogenase (G3PDH) function [22,23], which results in the accumulation of glycolytic metabolites. This subsequently stimulates the synthesis of polyol, hexosamine, and diacylglycerol (DAG), the activation of the protein kinase C (PKC) pathway, and the production of AGEs [24]. The interaction between AGEs and their RAGE receptors further promotes the overproduction of ROS and the activation of NF-κB, which then upregulates the expression of inflammation-related genes, leading to increased levels of interleukin (IL)-6, tumor necrosis factor-α (TNF-α), and monocyte chemoattractant protein-1 (MCP-1) [25,26,27]. Coupled with the oxidative stress, endoplasmic reticulum (ER) stress, and inflammatory processes induced by high glucose levels, the availability of nitric oxide (NO) is reduced and angiogenesis is impaired, which can lead to endothelial dysfunction in the kidneys [28].

Activation of the hexosamine pathway by high glucose levels can influence signal transduction, gene transcription, cell survival, and proteasome-mediated degradation, and promote hyperglycemia-induced vascular damage [29]. It is known that high blood glucose promotes the accumulation of extracellular matrix (ECM) [30] and upregulates the expression of DKK1, the Kremen-2 receptor, transforming growth factor-beta (TGF-β), and fibrotic factors in mesangial cells [31], which ultimately escalates to damage to the glomerular filtration barrier to cause DKD.

DKD and DM patients are known to have an enhanced susceptibility to adverse cardiovascular outcomes, partly due to activation of the renin–angiotensin–aldosterone system (RAAS). RAAS regulates blood pressure, salt balance, and fluid homeostasis [32], and RAAS blockade with ACE inhibitors (ACEI) or angiotensin receptor blockers (ARB) is often used to modify hyperfiltration states and delay progression of renal disease [33]. Drugs that control hypertension (lisinopril) and hyperglycemia (empagliflozin) were also shown to improve the physiological and histopathological features of kidney disease in a mouse model of hypertension-accelerated progressive DKD [34]. Moreover, treatment with N-acetyl-seryl-aspartyl-proline (Ac-SDKP), a naturally occurring immunomodulatory and angiogenic peptide mainly produced through enzymatic hydrolysis involving meprin-α and prolyl oligopeptidase, has been shown to partially improve end-organ damage by reducing inflammation and fibrosis, and promoting angiogenesis [35]. The beneficial effects of the selective mineralocorticoid receptor antagonist (MRA) eplerenone on renal outcome parameters such as proteinuria have been noted for some time, and efforts are being made to develop MRAs as adjunctive therapies to reduce the risk of DKD [36,37]. Dipeptidyl peptidase-4 (DPP-4) inhibitors commonly used for the treatment of type 2 diabetes have also been shown to prevent diabetic renal injury via various mechanisms; for example, inhibition of DPP-4 by linagliptin reduced obesity-related insulin resistance and inflammation through the regulation of M1/M2 macrophage status, and was further able to alleviate oxidative stress and diabetic renal injury [38].

Glycosuria induces osmotic diuresis, and commonly occurs in DM patients when the amount of filtered glucose exceeds the capacity of renal tubular reabsorption. SGLT2 inhibitors are a class of medications that alter the essential physiology of the nephron, and can lower blood sugar by inducing the kidneys to remove sugar from the body through the urine. SGLT2 inhibitors can also help to restore function to SIRT3, a mitochondrial NAD^+^-dependent deacetylase that can inhibit epithelial–mesenchymal transition (EMT) and renal fibrosis [39], and which is suppressed by high glucose levels. Treatment with inhibitors that block acetylation-mediated STAT3 binding has also been shown to reduce proteinuria and kidney damage in db/db diabetic mouse models.

## 3. Genetic Pathways Associated with DKD

**Figure 1:**
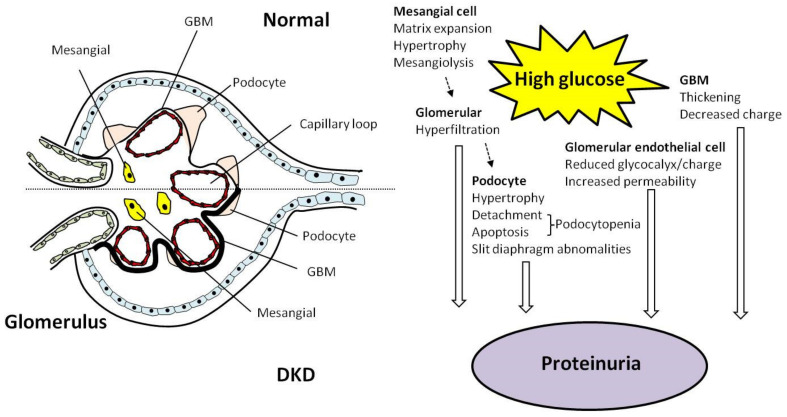
Characteristic glomerular changes and mechanisms of proteinuria in diabetic kidney disease. Characteristic glomerular changes in diabetic kidney disease (DKD) include glomerular basement membrane (GBM) thickening and mesangial expansion (due to increased mesangial matrix and increased mesangial cell size caused by hypertrophy). These changes are driven by hyperglycemia, and can ultimately lead to proteinuria if left unaddressed. Dashed arrows indicate mesangial expansion leading to glomerular hyperfiltration.

Glomeruli are the basic filtering units of the kidney, and consist of capillary blood vessel structures that can filter plasma and form urine [40]. Each glomerulus contains mesangial cells, podocytes, tubular cells, and the basement membrane, all of which act together to maintain normal filtration functions (Figure 1). Mesangial cells account for 30–40% of all cells in a glomerulus [41], and are responsible for removing immune complexes and protein aggregates from blood trapped in the basement membrane [42].

Podocytes are highly specialized epithelial cells that cover the outer surface of the basement membrane [43], and in adults, they are terminally differentiated and do not replicate. Consequently, more than 20% loss of podocytes or impairment of the glomerular filtration barrier structure can irreversibly damages a glomerulus and lead to proteinuria [44]. It is known that hyperglycemia can cause apoptosis, detachment of the glomerular basement membrane, and the loss of glomerular podocytes, mesangial hypertrophy, matrix accumulation, and basement membrane thickening, all hallmarks of early DKD [45], and can ultimately progress to glomerular fibrosis and proteinuria [46] (Figure 1).

### 3.1. The Role of Hyperglycemia in Glomerular Fibrosis

Long-term injury and abnormal wound healing processes, as well as excessive extracellular matrix deposition, constitute the main drivers of renal fibrosis. Myofibroblasts are thought to be the primary activated fibroblast phenotype in renal fibrosis [47], and there are several known sources of myofibroblasts that produce matrix, including activated residential fibroblasts, differentiated pericytes, recruited circulating fibroblasts, and mesenchymal cells transformed from macrophages, derived from renal tubular epithelial cells through EMT, or transformed from endothelial cells (EC) through endothelial–mesenchymal transition (EndMT) [48]. In addition, inflammatory cells and cytokines, as well as the associated signaling pathways, all play major roles in fibroblast activation [49].

Dense fibrosis of the glomerular microenvironment, particularly mesangial cells, is a hallmark of DKD, and the fibrosis of mesangial cells is closely associated with activation of the TGF-β1 signaling pathway, which promotes fibroblast activation and the abnormal synthesis of fibrotic matrix in mesangial cells [50]. In addition, TGF-β1 promotes renal cell proliferation and differentiation, synthesis of extracellular matrix [51], and renal tubular epithelial cell EMT, which is essential for the development of tubulointerstitial fibrosis [52,53]. It is likely that these adverse changes in mesangial cells, endothelial cells, and podocytes may be interrelated, and hyperglycemia is also known to disrupt the podocyte–glucocorticoid receptor signaling pathway to trigger EndMT and cause glomerular fibrosis in DM.

Glomerular fibrosis is also associated with the activation of Wnt/β-catenin signaling, which modulates TGF-β1-mediated fibrosis in mesangial cells [54] and can activate glycogen synthase kinase-3β (GSK-3β) signaling [55] and induce the apoptosis of mesangial cells [56]. An earlier study showed that GSK-3β phosphorylation decreased fibroblast activation and development of fibrosis in mice, but Wnt/β-catenin signaling acted to inhibit this [57]. Alternately, the inhibition of Wnt signaling by DKK1 reduced β-catenin phosphorylation and attenuated TGF-β1 expression to decrease the fibrosis of mesangial cells [31].

Another factor involved in DKD is the cannabinoid receptor 1 (CB1R) [55], which activates the expression of the hormone receptor peroxisome proliferator-activated receptor γ2 (PPARγ2); subsequent binding of adipocyte-specific nuclear hormones to PPARγ2 then activates the transcription of genes involved in adipogenesis, including *aP2*, *FGF1*, *FGF21*, and *CD36* [56], and promotes insulin sensitization in lipid metabolism [56]. An earlier study showed that overexpression of PPARs is closely related to metabolic syndrome, resulting in changes of lipid metabolism and accumulation of body fat, which induce DKD and increase disease severity [57]. Under hyperglycemic conditions, CB1R is known to adversely affect metabolism and increase insulin resistance to exacerbate DKD. CB1R also promotes the expression of proteins that are associated with kidney fibrosis to worsen DKD, including those that activate Ras and ERK signaling, transcription factor c-Jun, inflammation regulator SOCS3, and the proinflammatory cytokines IL-1β and fibrotic matrix fibronectin [58].

### 3.2. Hyperglycemia-Induced Glomerular Dysfunction and Proteinuria

Proteinuria is a condition of increased protein levels in the urine, and is a sign of kidney damage. It is known that hyperglycemia-induced mitochondria fission heightens the production of ROS to cause proteinuria and promote apoptosis in podocytes and kidney microvascular endothelial cells [59,60]. This process is mediated by dynamin-related protein-1 (Drp1) [59], and a previous study showed that Drp1 translocation into the mitochondria is mediated via phosphorylation and recruitment by Rho-associated coiled-coil containing protein kinase 1 (ROCK1) [59], thus explaining why the expression of ROCK1 in diabetic mice promotes glomerular apoptosis and mitochondrial ROS production. Additionally, hyperglycemia-induced expression of renal hedgehog interacting protein (Hhip) in glomerular endothelial cells may contribute to the fibrosis and apoptosis of such cells [61], and Hhip levels are also elevated in early DKD of diabetic mice and humans, even before the development of microalbuminuria [62]. Besides mitochondrial fission, activation of the Notch signaling pathway is known to promote the development of glomerular diseases, including proteinuria. A study showed that the intracellular domain of Notch1 activates vascular endothelial growth factor (VEGF) to induce podocyte apoptosis and cause proteinuria [63]. A previous study also showed that inhibition of this pathway protects rats with proteinuria [64].

Other signaling pathways associated with proteinuria include the Wnt/β-catenin pathway; elevated expression of Wnt/β-catenin transcripts and proteins has been observed in the podocytes of DKD patients and DKD mouse models, while stable expression of Wnt/β-catenin genes in the podocytes of transgenic mice was shown to induce albuminuria [65]. In addition, hyperactivation of mTOR is known to induce podocyte hypertrophy and apoptosis of podocytes, which aggravates glomerular disease and proteinuria [66,67]. It is also known that reduced nephrin expression is involved in hyperglycemia-induced albuminuria [68,69]. Nephrin is a transmembrane protein with extracellular domains that connect the foot processes of podocytes, and is essential for the proper functioning of the renal filtration barrier. Podocytes are known to have a complex actin cytoskeleton architecture, and redistribution of the actin cytoskeleton and disruption of this architecture is known to decrease nephrin expression [70]; for example, Rac1 and Cdc42 are known to regulate the dynamics of the actin cytoskeleton [71], and deletion of their genes was observed to reduce nephrin expression and induce albuminuria in mice [72].

### 3.3. Hyperglycemia and Albuminuria in Renal Tubular Cell Fibrosis

DKD is closely associated with the fibrosis of renal tubular epithelial cells [73], which are epithelial cells located at the outer layer of the renal tubule that act to reabsorb glucose, amino acids, and other substances in the urine [74]. An earlier study showed that exposure to high glucose or albumin levels can induce renal tubular epithelial cell fibrosis, and this was closely associated with the increased expression of MCP-1, PAI-1, and TGF-β1 as a result of hyperglycemia-induced ROS production [75]; renal fibrosis can be prevented if these profibrosis genes are suppressed [76]. In addition, the fibrosis of renal tubular epithelial cells is closely related to albuminuria, which in turn activates the unfolded protein response [77] to induce apoptosis [78]. The inhibition of apoptosis may increase autophagy in tubular epithelial cells [79], leading to worsened inflammation and fibrosis [80,81]. Hyperglycemia can also cause renal tubular epithelial cells to lose their polarity and acquire migration and invasive properties [82], leading to increased expression of fibronectin and α-smooth muscle actin (α-SMA) and decreased expression of E-cadherin to cause fibrosis.

### 3.4. Endothelial Cell Dysfunction in Diabetes-Related Renal Fibrosis

**Figure 2:**
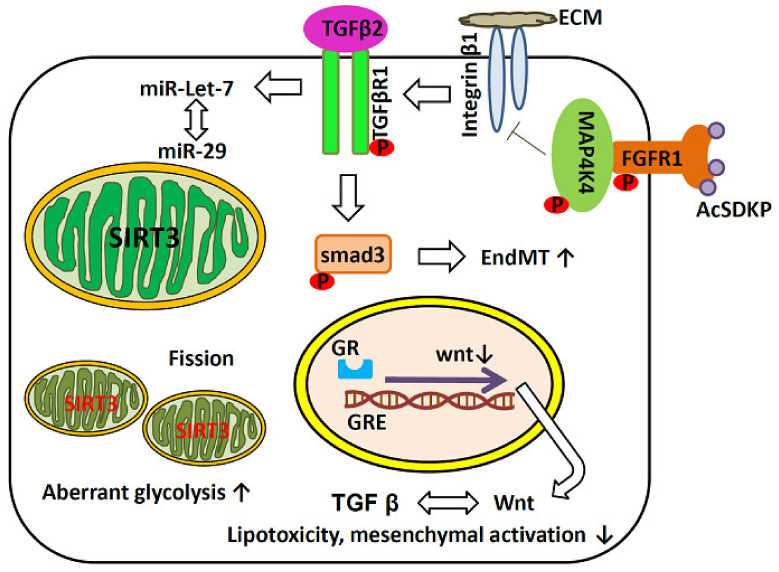
Endothelial cell dysfunction in renal fibrosis. AcSDKP: N-acetyl-seryl-aspartyl-proline; ECM: extracellular matrix; EndMT: endothelial-to-mesenchymal transition; FGFR: fibroblast growth factor receptor; GR: glucocorticoid receptor; GRE: glucocorticoid response element; TGF-β: transforming growth factor-β; MAP4K4: mitogen-activated protein kinase kinase kinase kinase 4. Red SIRT3 indicates deficiency and black SIRT3 indicates sufficiency. ↑: Increase in the expression level; ↓: Decrease in the expression level.

Fibrosis is characteristic of progressive chronic kidney diseases of any etiology, and eventually leads to kidney failure (Figure 2). Recently, several new signaling molecules that regulate renal fibrosis have been reported. Glucocorticoid receptor (GR) is a nuclear hormone receptor that mediates steroid hormones and is commonly expressed in most cell types, including the kidney. The role of glucocorticoids in cardiovascular and renal diseases is complex. Endothelial GR is a negative regulator of vascular inflammation in models of sepsis and atherosclerosis [83,84]. Loss of endothelial GR can induce upregulation of the Wnt signaling pathway, which in turn promotes renal fibrosis [85]. Thus, endothelial GR is an essential antifibrotic molecule in diabetes.

Transgenic mice with reduced STAT3 activation ability show less proteinuria, mesangial expansion, cell proliferation, macrophage infiltration, inflammation, and abnormal matrix synthesis when treated with streptozotocin for diabetes [86]. Mitochondrial SIRT3 is a NAD^+^-dependent deacetylase, which mainly exerts antioxidant activity to prevent aging-related diseases [87]. SIRT3 deficiency can lead to impaired insulin secretion, renal fibrosis, increased mitochondrial protein acetylation, and increased mitochondrial oxidative stress [88]. SIRT1 uses cellular NAD^+^ to deacetylate a variety of proteins involved in mitochondrial biogenesis, oxidative stress, inflammatory apoptosis, and autophagy. Inhibition of acetylation-NF-κB via activation of SIRT1 improves kidney inflammation in diabetic mice [89]. Under hyperglycemic conditions, the downregulation of AMPK/SIRT1/PGC-1α induces hypertrophy, ROS, and mitochondrial and autophagy dysfunction, all of which promote the development of DKD. AMPK upregulates SIRT1 by increasing cellular NAD^+^ levels [90], and both AMPK and SIRT1 have been identified as intracellular energy sensors, which, respectively, detect and respond to AMP/ATP and NAD^+^/NADH ratios, and are activated under energy expenditure conditions and inactivated in DM [91].

It is also known that FGF (fibroblast growth factor) signaling maintains endothelial barrier function and endothelial cell survival via binding with related FGFR [92]. The AcSDKP–FGFR1–MAP4K4 axis has an important role in combating EndMT-associated fibrotic disorders [93] and, as the target of AcSDKP, endothelial FGFR1 is essential as an antifibrotic core molecule [94].

## 4. Abnormal Transcriptional Regulation Leads to DKD

Transcriptional regulation is critical to the maintenance cellular homeostasis. However, hyperglycemia is known to transcriptionally induce the expression of specific genes, which become constitutively expressed even after hyperglycemia is controlled, and this can contribute to kidney damage in DKD patients [95]. This section describes how transcriptional regulation influences DKD.

### 4.1. Dysregulation of Transcription Factors and DKD

Transcription factors bind to specific sequences in promoters to regulate transcription, and, under high-glucose conditions, many signal transduction pathways are activated to regulate transcription, which in turn can influence the development of DKD. It is well known that Wnt signaling is critically involved in podocyte fibrosis [96]; for instance, high glucose is known to activate the Wnt signal transduction pathway, leading to the phosphorylation of β-catenin. Phosphorylated β-catenin then activates the transcription of Snail1, MMP-7, and Fsp1, and promotes podocyte dedifferentiation and mesenchymal transformation to cause podocyte fibrosis [97]. Caudal-type homeobox transcription factor 2 (CDX2) can activate the transcription and expression of cystic fibrosis transmembrane conductance regulator (CFTR) to suppress Wnt signaling and prevent fibrosis [98], and an early study showed that expression of CDX2 improved renal tubular lesions in DKD patients and a mouse DKD model [98].

ROS play an important role in tubulointerstitial fibrosis caused by the activation of myofibroblasts [99]. An antioxidative transcription factor, NF-E2-related factor 2 (NRF2), is known to activate the transcription of glutathione peroxidase 2 (GPX2) to increase oxidative stress, inflammation, and apoptosis, leading to permanent injury with renal fibrosis and DKD [100]. NRF2 is expressed constitutively; however, it is degraded by the NRF2-Kelch-like ECH associated protein 1 (Keap1) via the ubiquitin–proteasome pathway [101]. As Keap1 contains reactive cysteine residues that can form adducts with oxidants and electrophiles to sense cellular oxidative stress, NRF2 is stabilized under oxidative stress conditions. NRF2 plays a central role in protecting renal cells from oxidative injury by activating the genes encoding glutathione and NADPH to combat oxidative stress [102], and can further activate the pentose phosphate pathway through the production of NADPH, which may be associated with renoprotection from oxidative damage [102].

FoxO1 is another transcription factor that is closely associated with DKD. Many genes regulated by FoxO1 are known to prevent renal tubulointerstitial fibrosis and apoptosis, both of which play important roles in the pathogenesis of DKD [103]. It is known that high glucose promotes FoxO1 phosphorylation in kidneys [104] to activate the transcription of genes involved in gluconeogenesis and glycogenolysis, thereby causing proteinuria and renal fibrosis [105]. Inhibition of the function of FoxO1 by natural compounds or synthetic drugs was shown to attenuate renal cell damage in a high-glucose environment [106]. Dachshund homolog 1 (DACH1) is another transcription factor that is related to DKD. DACH1 recruits Pax transactivation-domain interacting protein (PTIP) to repress transcription in podocytes; this requires DACH1 sequence-specific DNA binding and reduces methylation of histone H3 at K4 to activate the transcription of *NELL2* and increase podocyte injury [107].

### 4.2. Influence of Genes Regulated by Krϋppel-Like Factors in DKD

Krϋppel-like factors (KLFs) are a group of transcription factors that include at least 27 proteins. Many of these KLF members, including KLF2, KLF4, KLF5, KLF6, and KLF15, are known to activate genes in glomerular endothelial cells or podocytes to prevent fibrosis; although KLF 10 appears to have a deleterious effect on the kidney [108,109,110,111,112,113,114,115]. The involvement of KLFs in DKD is detailed in the following subsections.

#### 4.2.1. Renoprotective Effect of KLFs

KLF2 activates a tight junction protein, occludin, to prevent the formation of gaps between endothelial cells and maintain the integrity of the endothelial barrier [116]. Under high-glucose conditions, the expression of KLF2 is repressed by FoxO1 [117], which causes glomerular endothelial cell and podocyte damage [113].

KLF4 expression reduces GpC methylation at the nephrin promoter and the promoters of other epithelial markers [111] to protect the kidneys under normal conditions, but high glucose levels have been observed to reduce KLF4 messenger RNA levels and increase the expression of macrophage migration inhibitory factor (MIF) and MCP-1, in a process mediated by TGF-β1 and typically suppressed by KLF4 [118]. TGF-β1 is a key driver of renal fibrosis, and the expression of TGF-β1 promotes the development and progression of renal disease [119] while also activating the expression of Twist1 or Snail to prolong G2/M arrest and promote renal fibrosis [120]. KLF-4 acts to suppress the cell proliferation and differentiation induced by TGF-β1 [121]. In addition, KLF5 significantly attenuates the expression of Bax, caspase-3, caspase-8, and caspase-9 in podocytes [122] by blocking the activation of mitogen-activated protein kinase (MAPK) pathways [122,123]. A previous study confirmed that regulating P38-induced apoptosis [124] and inhibiting apoptosis via MAPK pathways could be an effective strategy to reduce renal fibrosis [125].

Cytochrome c-oxidase (COX) plays a key role in the regulation of aerobic energy production via the mitochondrial respiratory chain. In podocytes, KLF6 regulates mitochondrial function through the COX assembly gene (SCO2), which modulates the balance between mitochondrial respiration and glycolytic pathways to prevent mitochondrial dysfunction and podocyte apoptosis [109]. Additionally, KLF15 inhibits TGF-β1 through the ERK/MAPK and JNK/MAPK pathways [126], and is a key regulator of podocyte differentiation and a protector against podocyte damage [127].

#### 4.2.2. KLF10 Causes Kidney Damage in DKD

**Figure 3:**
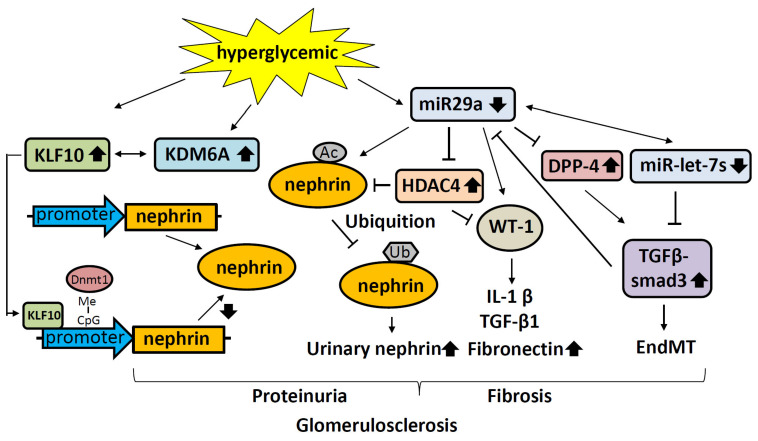
Hyperglycemia-induced nephrin modification induces glomerulosclerosis. Ac: acetylation; Dnmt1: DNA methyltransferase 1; EndMT: endothelial-to-mesenchymal transition; HDAC4: histone deacetylase 4; IL-1β: Interleukin-1β; KDM6A: lysine-specific demethylase; KLF 10: Krϋppel-like factor 10; Me: methylation; TGF-β: Transforming growth factor β; Ub: ubiquitination; WT1: Wilms’ tumor 1 protein. ↑: Increase in the expression level; ↓: Decrease in the expression level.

KLF10 has multiple roles in podocyte dysfunction and injury. TGF-β1, bone morphogenetic protein-2 (BMP-2), and epidermal growth factor (EGF) induction of KLF10 expression play an important role in the transcription of genes such as Smad, which is involved in cell proliferation, apoptosis, and differentiation [128]. KLF10 also inhibits nephrin expression through interaction with DNA methyltransferase 1 (DNMT1) to methylate the nephrin promoter [115] (Figure 3). In addition, KLF10 represses the transcription of many genes specifically expressed in podocytes, including those encoding Wilms’ tumor 1 protein (WT1), podocin, synaptophysin, and nephrin, ultimately activating the expression of lysine-specific demethylase (KDM6A), which is essential to the maintenance of kidney function as a regulator of podocyte differentiation [129], to promote global epigenetic reprogramming and cause aberrant gene expression [115]. Finally, KLF10-induced expression of KDM6A induces proteinuria and irreversible kidney damage under diabetic conditions [115].

## 5. Epigenetic Modifications and the Pathogenesis of DKD

Acetylation of histones H3 and H4 is known to reduce the positive charge of chromatin, making promoters accessible to transcription factors for transcriptional activation [130]. Conversely, deacetylation has a reverse effect that results in transcriptional repression [131]. These processes are respectively catalyzed by histone acetyltransferase (HAT) and histone deacetylase (HDAC) [132]. Histone methylation and demethylation by methyltransferase and demethylase of the CpG islands in promoters are also known to respectively repress and activate gene expression [133].

### 5.1. DNA Methylation Is Associated with DKD

DNA methylation is a repressive epigenetic modification and has been implicated in the pathogenesis of DKD. Methylation of cytosine in the CpG islands of promoters is associated with transcriptional repression [134], and a microarray analysis of cytosine methylation in human kidney tubules revealed that kidney structural damage changes cytosine methylation and the degree of kidney fibrosis [135]. Another study also found that the expression of DNMT1 was elevated in peripheral blood mononuclear cells in DKD patients; the increase of DMNT1 can activate the mTOR pathway and inflammation [136].

The *RASAL1* gene encodes an inhibitor of the Ras protein and hyperglycemia is known to cause hypermethylation of *RASAL1*, which is associated with the perpetuation of fibroblast activation and renal fibrosis [137]. TGF-β1 is known to promote the expression of DNMT1 and DNMT3 to stimulate hypermethylation and repress the transcription of *RASAL1*, resulting in the activation of fibrogenesis [138,139,140]. Another protein that is known to be associated with renal fibrogenesis is fibronectin [141]. After renal injury, a healing process begins, and fibronectin in the ECM is the first protein that is deposited and accumulated during fibrogenesis [141]. The accumulation of fibronectin is closely associated with fibrosis [141], and an earlier study showed that methylation levels of the *MMP9* gene promoter in DKD patients were reduced, leading to elevated expression of fibronectin [142]. Hypomethylation of inhibitors of *MMPs (TIMP-2)* and *AKR1B1* genes, which encode aldose reductase, are also associated with proteinuria in patients with early DKD [143].

### 5.2. DKD Is Associated with Post-Translational Modification of Histones

Histone acetylation is known to relax chromatin structure and facilitate the binding of transcription factors to promoters to activate transcription. Conversely, methylation of histones has an opposite effect, repressing transcription. Hyperglycemia often influences these processes to cause kidney disorders [144].

#### 5.2.1. Histone Acetylation Is Involved in DKD Pathogenesis

Histone acetylation is involved in the progression of DKD [145,146,147], and an earlier study showed that acetylation of H3K9 was elevated in the kidneys of DKD patients [145]. Meanwhile, acetylation of histones H3 and H4 is known to activate the transcription of Cola1, CTGF, PAI-1, P21, Lacm1, FN1, TNF-α, COX-2, and MCP-1 [148], which can promote DKD development. In addition to histone acetyltransferase, histone deacetylases (HDACs) regulate gene expression epigenetically by removing acetyl groups from histones to repress transcription, and this can promote the development of DKD as well. For instance, the expression of nephrin, which protects podocytes from damage caused by hyperglycemia, is repressed by HDAC4. An earlier study also showed that the expression of nephrin was elevated following miR-29a, which is known to reduce the expression of HDAC4 and result in elevated expression of nephrin [149] (Figure 3).

Myofibroblastic differentiation is a process that produces terminally differentiated myofibroblasts and is involved in tissue healing [150]. Myofibroblasts accumulate interstitial ECM components such as collagens and fibronectin during wound healing, and express abundant amounts of smooth muscle α-actin (α-SMA) [151,152]. These cells are ultimately incorporated into stress fibers [152]. It is well documented that TGF-β1 mediates the turnover of the ECM to promote myofibroblastic differentiation in the kidney. An earlier study showed that HDAC4 is required for TGF-β1-induced myofibroblastic differentiation, as inhibiting histone deacetylation by trichostatin A or silencing the expression of HDCA4 inhibited the transcription of the α-SMA gene [153], revealing a critical role for histone acetylation in renal fibrosis. Additionally, SIRT3, a histone deacetylase in the mitochondria, influences glycolysis and fibrosis through the regulation of PKM2 dimer and HIF1α levels [154], and also mediates STAT3 phosphorylation to affect aberrant glycolysis in tubules.

#### 5.2.2. Histone Methylation Is Involved in DKD Pathogenesis

Histone methylation has monomethyl, dimethyl, and trimethyl forms, and the extent of histone methylation is known to modulate gene transcription [148] and influence DKD pathogenesis [155]. SUV39H1 is a histone methyltransferase that catalyzes methylation of the K9 residue in H3 with dimethyl or trimethyl groups. Hyperglycemia has been shown to decrease the expression of SUV39H1 to promote renal fibrosis [156,157]. Earlier studies demonstrated that the development of DKD is associated with elevated transcription of pro-inflammatory or profibrotic genes, due to decreased methylation of histone H3 at the promoters of these genes [147,158,159]. It well known that p21WAF1 is transcribed at high levels after acute kidney injury [160], and inhibition of methyltransferase SUV39H1 expression has been found to attenuate hyperglycemia-induced fibronectin and p21WAF1 expression, and to accelerate hyperglycemia-induced cell hypertrophy [161]. Attenuation of SUV39H1 expression in turn suppresses high-glucose-induced expression of fibronectin and p21WAF1 [161]. The overexpression of SUV39H1 and H3K9 methylation in DKD patients has been noted to reduce kidney inflammation and cell apoptosis [162].

### 5.3. Noncoding RNA Is Involved in DKD Pathogenesis

Noncoding RNA is also involved in the progression of DKD inflammation and fibrosis [163] (Table 1), and it is known that long noncoding RNAs (lncRNAs) participate in the initiation and progression of DKD by exerting direct pathogenic effects, or by indirectly mediating specific renal pathways (such as TGF-β1, NF-κB, STAT3, and GSK-3β signaling) [164]. Thus, lncRNAs may have potential as biomarkers for the early diagnosis or prognosis tracking of DKD, or as therapeutic targets for slowing the progress or even reversing established DKD. High-glucose conditions have been shown to increase the expression of miR-34a, which induces mesangial proliferation and glomerular hypertrophy through the inhibition of growth arrest-specific 1 (GAS1) [165]. GAS1 is involved in glomerular cell proliferation and activation, and is expressed in the kidney under pathological conditions [166]. Additionally, inhibition of miR-196a expression induces mesangial hypertrophy by activating the cyclin-dependent kinase inhibitor p27kip1, thereby preventing cell-cycle arrest in the G1 phase [167]. The expression of miR-93 further increases expression of mitogen and stress-activated kinase 2 (Msk2), which in turn mediates chromatin remodeling and podocyte gene transcription to cause DKD [168].

As discussed previously in this article, CB1R is expressed in the kidney, and activating of CB1R expression by hyperglycemia is known to cause renal injury and nephropathy [149]. In a transfection system, miR-29a was found to suppress the expression of CB1R in the mesangial cells of high-glucose-stressed mice, thus blocking the expression of pro-inflammatory and profibrotic mediators to attenuate renal hypertrophy [169]. Furthermore, curcumin is known to have beneficial effects on reducing the severity of DKD, as this natural compound promotes the expression of miR-29a to inhibit CB1R [170]. In contrast, decreasing the expression of miR-29a attenuates DKK-1/Wnt/β-catenin signaling and promotes apoptosis and ECM deposition to influence kidney fibrosis [31]. Additionally, miR-29c is known to activate Rho kinase by targeting Spry-1, which is related to ECM accumulation. Moreover, podocyte apoptosis is known to be controlled by miR-29c and miR-21, and when miR-29c expression increases, it promotes fibronectin assembly and apoptosis [171]. The noncoding RNA miR-let-7 decreases ECM protein expression through a mechanism that involves the TGF-β/Smad3 pathway [172], and DPP-4 inhibition and promotion of peptide AcSDKP expression result in renal protection by regulating crosstalk between miR-29 and miR-let-7 [173].

EMT and EndMT processes play vital roles in the development of fibrosis in the kidney. The noncoding RNA miR-21 is a downstream target of Smad3, which is known to activate the transcription of miR-21 in the presence of TGF-β [174]; miR-21 also inhibits pro-apoptotic signals and ameliorates glomerular injury induced by TGF-β and hyperglycemia [175]. TGF-β1 is known to induce signal circuit amplification and activation of a chronic state of profibrosis, and can regulate the expression of miR-192, the miR-200s, miR-21, and miR-130b in mesangial cells [176,177,178]. Meanwhile, miR-93 is involved in TGF-β1-induced EMT and renal fibrogenesis [179], while downregulation of miR-23a inhibits high-glucose-induced EMT and renal fibrogenesis [180]. Studies show that miR-192 levels are enhanced in glomeruli isolated from streptozotocin-injected diabetic mice, as well as diabetic db/db mice [181]. In addition, the miR-200s are enriched in the kidney, and expression of miR-200s is stimulated by oxidative stress. The miR-200s have been shown to regulate mesenchymal-to-epithelial transition (MET) through modulation of the E-cadherin transcriptional repressor zinc finger E-box binding homeobox 1 (ZEB1) [182]. However, the miR-130b-SNAIL axis acts to promote EMT and progression toward increased tubulointerstitial fibrosis in DKD [183].

High glucose further promotes the expression of TGF-β1 to activate the expression of miR-377, which suppresses the expression of p21-activated kinase (PAK) and superoxide dismutase (SOD), thereby enhancing fibronectin protein production [184]. TGF-β1 reduces the expression of antifibrotic miRNAs (miR-29s and let-7) [185,186], which target different collagen isoforms in mesangial cells. Other protective miRNAs include miR-26a, which inhibits TGF-β1-induced ECM protein expression in DKD patients [187], and miR-146a, which is upregulated in early DKD to reduce the expression of inflammatory cytokines such as IL-1β and IL-18 [188].

## 6. Conclusions

**Figure 4:**
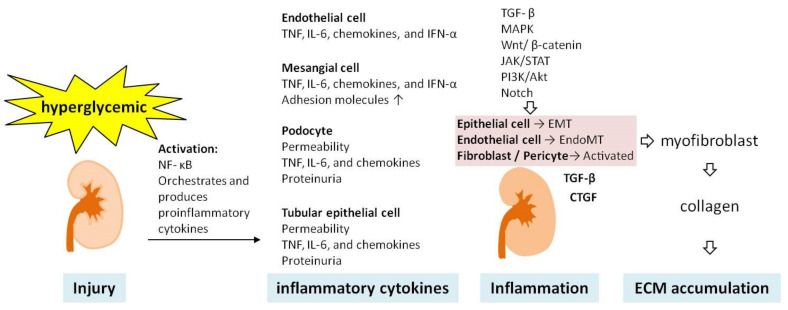
Mechanisms driving renal inflammation and fibrosis. ECM: extracellular matrix; EMT: epithelial–mesenchymal transition; EndoMT: endothelial–mesenchymal transition; IFN-α: interferon alpha; IL-6, interleukin 6; JAK/STAT: Janus kinase signal transducers and activators of transcription; MAPK: mitogen-activated protein kinase; TGF-β: transforming growth factor-beta; TNF, tumor necrosis factor.

Hyperglycemia is mediated by cytokines, growth factors, and nonepigenetic mechanisms, the latter of which are also some of the key drivers of DKD, involving Wnt/β-catenin signaling, ER stress, excessive ROS, RAAS activation, albumin overload, and the additional production of inflammatory elements. However, epigenetic mechanisms such as DNA methylation, histone post-translational modifications, and noncoding RNAs also play critical roles in DKD pathogenesis, as do the processes of inflammation and fibrogenesis (Figure 4). It is well known that early-stage DKD can be improved through multidisciplinary treatment, but as the disease progresses, there is no effective treatment available as yet. The information provided in this review can assist clinicians in detecting DKD at an earlier stage, where it will be relatively easier to manage or slow the progression of nephropathy, and hopefully to achieve better outcomes.

## Figures and Tables

**Table 1:** Some miRNAs involved in diabetes-related renal inflammation and fibrogenesis.

Functions	miRNAs	Expression Levels	Target Genes	Mechanisms
Inflammation	miR-21	Upregulation	TIMP3	Enhanced the excretion of pro-inflammatory factors
	miR-146a	Upregulation	IRAK1/TRAF6	Promotes NF-κB mediated upregulation of pro-inflammatory cytokines
	miR-146a	Downregulation	Nox4	Decreases ROS generation and inflammation
	miR-29c	Upregulation	Spry-1	Activates Rho kinase by targeting Spry-1, related to ECM accumulation
Fibrosis	miR-192	Upregulation	GLP1R	Exerts pro-fibrotic effects
	miR-93	Downregulation	Orai1	Induces TGF-β1-induced EMT and renal fibrogenesis
	miR-29a	Downregulation	COL4A1, COL4A2, HDAC4, LAMC2	Increased the production of collagen IV protein by directly targeting the 3′UTR of *col4α1* and *col4α2*
	Let-7	Downregulation	HMGA2, IGF2BP2, TGFBR1, JAG1, THBS1	Decreases ECM protein expression through a mechanism that involves the TGF-β/Smad3 pathway
Both inflammation and fibrosis	miR-29b	Downregulation	SP1/Smad-3/NF-κB	Inhibition of Sp1 expression, TGF-β/Smad3-dependent renal fibrosis, and NF-κB-driven renal inflammation
	miR-199a-5p	Upregulation	Klotho	Activating the TLR4/NF-κB p65/NGAL signaling pathways and downstream fibrosis and inflammation
	miR-377	Upregulation	p21	Indirectly induces fibronectin by reducing the expression of p21- activated kinase and ROS

ECM: extracellular matrix; NF-κB: Nuclear factor-κB; ROS: reactive oxygen species; TGF-β: transforming growth factor-beta.

## Data Availability

Not applicable.
